# Sex differences in hospital outcomes of medically-managed type B aortic dissection

**DOI:** 10.3389/fcvm.2025.1597266

**Published:** 2025-05-08

**Authors:** Paulina Luna, Faris Amil, Mary J. Roman, Nickpreet Singh, Teagan Iranitalab, Jim W. Cheung, Ilhwan Yeo, Richard B. Devereux, Jonathan Weinsaft, Leonard Girardi, Alicia Mecklai, Rebecca Ascunce, Julie Marcus, Pritha Subramanyam, Amrita Krishnamurthy, Diala Steitieh, Luke Kim, Nupoor Narula

**Affiliations:** ^1^ Department of Internal Medicine, New York-Presbyterian Hospital, New York, NY, United States; ^2^ Division of Cardiology, Weill Cornell Medicine, New York, NY, United States; ^3^ Department of Medicine, Division of Cardiology, Weill Cornell Cardiovascular Outcomes Research Group (CORG), New York, NY, United States; ^4^ Department of Cardiothoracic Surgery, Weill Cornell Medicine, New York, NY, United States

**Keywords:** sex disparities, type B aortic dissection, readmissions, hospital outcomes, national readmissions database (NRD)

## Abstract

**Background:**

Medical management is recommended for uncomplicated type B aortic dissection (TBAD). However, data focused on sex differences in outcomes in TBAD patients managed medically are limited.

**Methods:**

Hospitalizations of adults with TBAD were identified using the 2016–2019 Nationwide Readmissions Database. TBAD diagnosis was deduced by inclusion of thoracic or thoracoabdominal aorta dissection and exclusion of presumed type A aortic dissection. Hospitalizations associated with intervention were excluded. Multivariable logistic regression modeling was used to investigate the association of sex with in-hospital mortality. A Cox proportional hazards model was used to assess the association between sex and readmission rates.

**Results:**

There were 52,269 TBAD hospitalizations (58% male). Compared to men, women were older (72 vs. 65 years), had higher in-hospital mortality (11.5% vs. 8.5%), shorter median length of stay (3.95 vs. 4.23 days), and lower rates of elective admissions (6.4% vs. 8.2%) (all *p* < 0.05). Despite similar rates of hypertension, lower rates of coronary artery disease and smoking, and decreased rates of hospital-related complications, women experienced increased adjusted in-hospital mortality (odds ratio: 1.16; 95% CI, 1.06–1.27). There were no sex differences in readmission risk at 30, 90, and 180 days.

**Conclusions:**

Women with TBAD managed medically experienced higher in-hospital mortality than men despite lower rates of atherosclerotic disease and risk factors. However, there were no sex differences in readmission risk at 30, 90, and 180 days. Prior research has demonstrated sex differences in presenting TBAD characteristics, including older age, varied signs/symptoms, and diagnostic delay in women. Data are needed to delineate additional causes of adverse acute outcomes in women managed medically, including condition- and medication-specific factors.

## Introduction

Aortic dissections affect 3–4 people per 100,000 each year (1) and are associated with high morbidity and mortality (2). Type B aortic dissections (TBAD) account for about 33% of all aortic dissections, with a male to female ratio of 1.5:1 (3–5). Medical management is recommended for uncomplicated TBAD while complicated TBAD — those associated with hypotension or shock, end-organ damage, refractory hypertension, neurologic sequelae, early aortic dilation or rupture — are managed with open surgical or thoracic endovascular aortic repair (TEVAR) (6, 7). Between 1996 and 2016, the majority of patients with TBAD were managed with medical therapy (57%–75%) while smaller proportions underwent endovascular management (7%–31%) and open surgery (8%–17%) (2, 8).

Prior studies have shown sex disparities in the management of patients with TBAD, with women being less likely to receive invasive procedures than men (3, 9, 10). However, in contrast to acute coronary syndromes (11, 12) and cardiogenic shock (13, 14), which are associated with worse outcomes in women compared to men, studies have shown that women with aortic dissections have variable outcomes (15–18). The majority of these studies have focused on patients with TBAD who are managed procedurally, have included those with type A aortic dissections, or have limited numbers (5, 17, 18). It is important to understand the scope of sex differences in patients with TBAD who receive medical therapy as they comprise the majority of patients with TBAD. Given the paucity of research focused on this patient cohort, this study investigates sex differences in readmissions and in-hospital outcomes in a large number of patients with TBAD managed medically.

## Methods

### Data source

This cross-sectional study sample was derived using data from the Nationwide Readmissions Database (NRD) from 2016–2019. The NRD is drawn from the Healthcare Cost and Utilization Project (HCUP) State Inpatient Databases (SID) and contains verified patient linkage numbers used to track patients across hospitals within states (19). The NRD contains data from approximately 18 million discharges each year (35 million total weighted discharges) across 30 states. These data include information on demographics, primary and secondary diagnosis/procedure codes based on International Classification of Diseases, Tenth Revision–Clinical Modification (ICD-10-CM) codes, length of stay (LOS), discharge disposition, death, admission cost-to-charge ratio, and hospital-level variables. Because data are de-identified, Institutional Review Board approval and informed consent were not required.

### Study population and variables

All hospitalizations of adults 18 years or older that were associated with dissection of thoracic or thoracoabdominal aorta were identified using ICD-10-CM codes I7100, I7101, and I7103. Given that there are no specific codes to distinguish between ascending and descending aortic dissection, hospitalizations associated with presumed type A aortic dissection were excluded using methods previously described (10, 20). Such hospitalizations included those associated with cardioplegia, valve repair, operation on the vessels of the heart, carotid or vertebral artery dissections, pericardial effusion, or aortic rupture. Hospitalizations associated with TEVAR or open repair were also excluded. The ICD-10-CM codes used for these exclusion criteria are listed in Supplementary Table S1.

Patient sociodemographic characteristics from the NRD included age, sex, insurance status, and median household income based on the patient's zip code. Race and ethnicity are not available in the NRD. Patient comorbidities were obtained from a combination of Elixhauser comorbidities and ICD-10-CM codes (Supplementary Table S1). Complications during hospitalization were also analyzed and included acute kidney injury (AKI), AKI requiring dialysis, stroke, mechanical ventilation, and cardiac arrest. The ICD-10-CM codes used for these outcomes are listed in Supplementary Table S1.

### Study outcomes

The primary outcome was in-hospital mortality and secondary outcomes included LOS and total charges.

### Statistical analyses

Analyses were performed using SAS version 9.4 (SAS Institute, Cary, NC, USA). Categorical variables were summarized as percentages and continuous variables were reported as medians and interquartile ranges, stratified by sex. Categorical variables were analyzed with the chi-squared test and continuous variables were evaluated using the nonparametric Wilcoxon rank-sum test given significant data skew. The NRD does not allow for year-to-year linkage of patients and hospitals, thus patients and hospitals from each year were considered as separate entities. Readmissions for any cause were considered within one calendar year or until death during hospitalization.

Multivariable logistic regression modeling was used to evaluate the association between sex and the above study outcomes. Cox proportional hazard models were constructed to assess the association between sex and readmission risk at 30, 90, and 180 days. The models were adjusted for patient sociodemographic factors and comorbidities.

Analyses accounted for the complex NRD survey design, which weighs admissions based on the stratification of hospitals by census region, ownership, location, and bed size. All statistical tests were two-sided, with *p* < 0.05 indicating statistical significance.

## Results

### Patient characteristics

**Figure 1:**
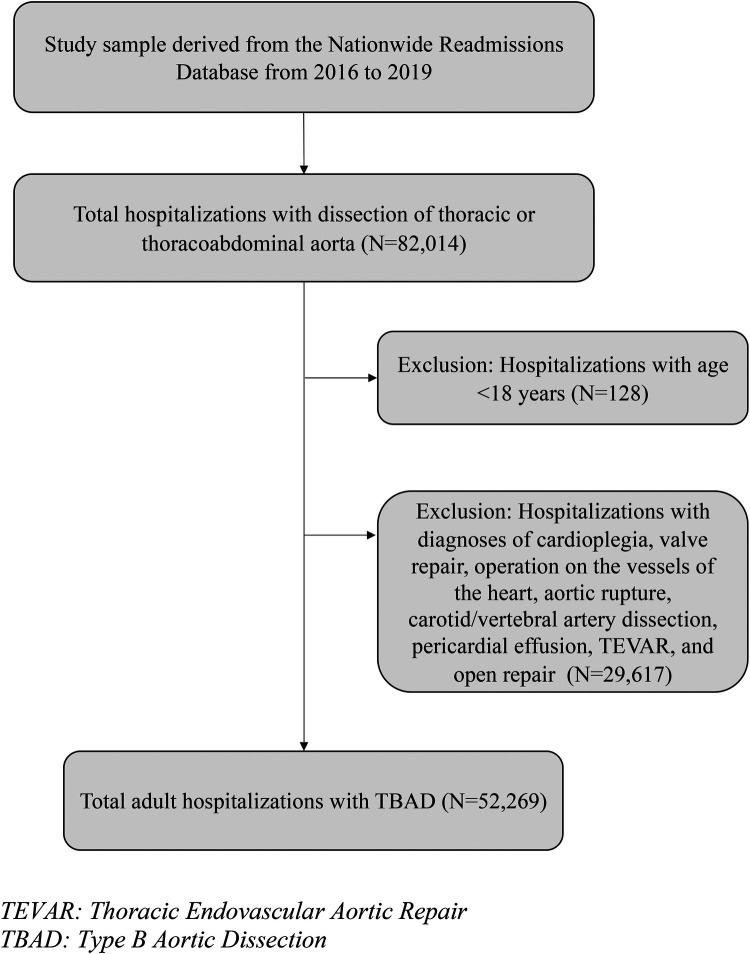
Flowchart of the analytic sample. TEVAR, thoracic endovascular aortic repair; TBAD, type B aortic dissection.

In total, there were 52,269 weighted hospitalizations of adults with TBAD (Figure 1). The majority of admitted patients were men (58%) and had a median age of 68 years (Table 1). Compared to men, women were older (median age 72 vs. 65 years), had higher rates of Medicare (70.8% vs. 54.5%), and had lower rates of elective admissions (6.4% vs. 8.2%; all *p* < 0.05). While women and men had similar rates of hypertension (83.7% vs. 84.2%, *p* = 0.28), women had lower rates of smoking (43.5% vs. 52.3%), coronary artery disease (35.5% vs. 38.2%), obesity (15.5% vs. 16.7%), and chronic kidney disease (28.5% vs. 33.0%; all *p* < 0.05). Women had higher rates of diabetes (22.9% vs. 19.9%) and dyslipidemia than men (45.5% vs. 42.7%; all *p* < 0.05). There was no statistically significant difference between women and men in rates of Ehlers-Danlos syndrome or Marfan syndrome (Table 1).

**Table 1:** Comparison of hospitalization characteristics stratified by sex.

Variable	Total Sample		Males		Females		*P*-Value
*N*=	52,269		30,099		22,170		
			(58%)		(42%)		
Demographics							
*Age, years, median (Q1, Q3)*	*68*	*(56, 79)*	*65*	*(54, 75)*	*72*	*(60, 83)*	*<0*.*0001*
*Insurance Status, n (%)*							*<0*.*0001*
* Medicare*	*32,078*	*61*.*4%*	*16,389*	*54*.*5%*	*15,689*	*70*.*8%*	
* Medicaid*	*6,751*	*12*.*9%*	*4,395*	*14*.*6%*	*2,356*	*10*.*6%*	
* Private Insurance*	*9,488*	*18*.*2%*	*6,382*	*21*.*2%*	*3,106*	*14*.*0%*	
* Self-pay*	*2,176*	*4*.*2%*	*1,597*	*5*.*3%*	*579*	*2*.*6%*	
Median Household Income, *n* (%)							0.32
Quartile 1 (lowest)	16,421	31.4%	9,507	31.6%	6,914	31.2%	
Quartile 2	13,368	25.6%	7,779	25.8%	5,589	25.2%	
Quartile 3	12,157	23.3%	6,906	22.9%	5,251	23.7%	
Quartile 4 (highest)	9,572	18.3%	5,422	18.0%	4,150	18.7%	
*Elective admission, n (%)*	*3,897*	*7*.*5%*	*2,476*	*8*.*2%*	*1,422*	*6*.*4%*	*<0*.*0001*
Comorbidities							
*Smoking, n (%)*	*25,379*	*48*.*6%*	*15,739*	*52*.*3%*	*9,640*	*43*.*5%*	*<0*.*001*
*Diabetes, n (%)*	*11,075*	*21*.*2%*	*5,994*	*19*.*9%*	*5,081*	*22*.*9%*	*<0*.*001*
*Coronary Artery Disease, n (%)*	*19,372*	*37*.*1%*	*11,500*	*38*.*2%*	*7,872*	*35*.*5%*	*<0*.*001*
*Dyslipidemia, n (%)*	*22,941*	*43*.*9%*	*12,860*	*42*.*7%*	*10,081*	*45*.*5%*	*<0*.*001*
*Chronic Lung Disease, n (%)*	*13,874*	*26*.*5%*	*7,000*	*23*.*3%*	*6,874*	*31*.*0%*	*<0*.*001*
*Liver Disease, n (%)*	*3,136*	*6*.*0%*	*2,106*	*7*.*0%*	*1,030*	*4*.*6%*	*<0*.*001*
*Chronic Kidney Disease, n (%)*	*16,268*	*31*.*1%*	*9,940*	*33*.*0%*	*6,329*	*28*.*5%*	*<0*.*001*
*Hypothyroidism, n (%)*	*6,871*	*13*.*1%*	*2,343*	*7*.*8%*	*4,528*	*20*.*4%*	*<0*.*001*
*Depression, n (%)*	*5,918*	*11*.*3%*	*2,569*	*8*.*5%*	*3,349*	*15*.*1%*	*<0*.*001*
*Drug Use Disorder, n (%)*	*2,912*	*5*.*6%*	*2,088*	*6*.*9%*	*824*	*3*.*7%*	*<0*.*001*
*Alcohol Use Disorder, n (%)*	*2,402*	*4*.*6%*	*1,945*	*6*.*5%*	*457*	*2*.*1%*	*<0*.*001*
*Heart Failure, n (%)*	*16,854*	*32*.*2%*	*9,475*	*31*.*5%*	*7,379*	*33*.*3%*	*0*.*01*
*Anemia, n (%)*	*17,616*	*33*.*7%*	*9,943*	*33*.*0%*	*7,673*	*34*.*6%*	*0*.*02*
*Obesity, n (%)*	*8,467*	*16*.*2%*	*5,022*	*16*.*7%*	*3,444*	*15*.*5%*	*0*.*03*
STEMI, *n* (%)	592	1.1%	314	1.0%	278	1.3%	0.13
Hypertension, *n* (%)	43,918	84.0%	25,355	84.2%	18,563	83.7%	0.28
Ehlers-Danlos Syndrome, *n* (%)	68	0.1%	32	0.1%	36	0.2%	0.35
Marfan Syndrome, *n* (%)	840	1.6%	506	1.7%	335	1.5%	0.42
Prior Myocardial Infarction, *n* (%)	4,289	8.2%	2,509	8.3%	1,781	8.0%	0.44
Peripheral Vascular Disease, *n* (%)	37,976	72.7%	21,920	72.8%	16,055	72.4%	0.50
Valvular Disease, *n* (%)	10,697	20.5%	6,194	20.6%	4,503	20.3%	0.62
Atrial Fibrillation, *n* (%)	13,792	26.4%	7,940	26.4%	5,852	26.4%	0.98

Italicized variables indicate statistical significance.

### TBAD management and outcomes

Table 2 depicts hospital complications stratified by sex. Rates of stroke (6.8% in women vs. 7.0% in men) and in-hospital cardiac arrest (2.0% in women vs. 2.2% in men) were similar between women and men. In addition, women overall had fewer hospital-associated complications, including lower rates of AKI (23.5% vs. 31.8%) and mechanical intubation (8.9% vs. 10.9%; all *p* < 0.05). Despite this, women had higher rates of in-hospital mortality than their male counterparts (11.5% vs. 8.5%, *p* < 0.05). After adjusting for patient characteristics, including age and comorbidities, female sex was associated with a higher likelihood of in-hospital mortality (OR 1.16, CI 1.06–1.27; Table 3). Women also had shorter median LOS (3.95 vs. 4.23 days) and lower total charges than men ($53,461 vs. $63,655), even after adjusting for in-hospital mortality (LOS: 4.20 vs. 4.33 days; total charges: $54,345 vs. $62,773; all *p* < 0.05). Women were more likely than men to be discharged to a skilled nursing facility (22.4% vs. 16.0%, *p* < 0.001) or home with home health care (23.1% vs. 17.9%, *p* < 0.001).

**Table 2:** Comparison of hospitalization complications and outcomes stratified by sex.

Variable	Total Sample		Males		Females		*P*-Value
*N*=	52,269		30,099		22,170		
*AKI, n (%)*	*14,795*	*28*.*3%*	*9,577*	*31*.*8%*	*5,218*	*23*.*5%*	*<0*.*0001*
*AKI Requiring HD, n (%)*	*1,052*	*2*.*0%*	*726*	*2*.*4%*	*327*	*1*.*5%*	*<0*.*0001*
*Intubation, n (%)*	*5,246*	*10*.*0%*	*3,281*	*10*.*9%*	*1,965*	*8*.*9%*	*<0*.*0001*
Stroke, *n* (%)	3,614	6.9%	2,117	7.0%	1,497	6.8%	0.39
Cardiac Arrest, *n* (%)	1,107	2.1%	660	2.2%	447	2.0%	0.36
*Died During Admission, n (%)*	*5,112*	*9*.*8%*	*2,558*	*8*.*5%*	*2,555*	*11*.*5%*	*<0*.*0001*
*Length of Stay, days, median (Q1, Q3)*	*4.10*	*(1.91, 7.87)*	*4.23*	*(1.96, 8.18)*	*3.95*	*(1.86, 7.48)*	*<0*.*0001*
*Total Charges, dollars, median (Q1, Q3)*	*59,069*	*(29,992, 124,291)*	*63,655*	*(31,917, 136,728)*	*53,461*	*(27,388, 108,001)*	*<0*.*0001*

Italicized variables indicate statistical significance.

AKI, acute kidney injury; HD, hemodialysis.

**Table 3:** Multivariate analysis of factors associated with in-hospital mortality.

Variable	Odds Ratio	95% Confidence Intervals	*P*-Value
Demographics			
*Age* ^a^	*1*.*04*	*1.03–1.04*	*<0*.*0001*
*Female Sex*	*1*.*16*	*1.06–1.27*	*<0*.*01*
*Elective Admission*	*0*.*48*	*0.37–0.61*	*<0*.*0001*
Insurance Status			
Medicare	1 [Reference]		
Medicaid	1.04	0.86–1.25	0.70
Private Insurance	1.01	0.86–1.18	0.91
*Self-pay*	*1*.*79*	*1.39–2.30*	*<0*.*0001*
Median Household Income			
Quartile 1 (lowest)	1 [Reference]		
Quartile 2	1.06	0.94–1.19	0.36
Quartile 3	0.99	0.87–1.12	0.82
Quartile 4 (highest)	1.09	0.97–1.24	0.16
Comorbidities			
*Hypertension*	*0*.*63*	*0.57–0.71*	*<0*.*0001*
*Peripheral Vascular Disease*	*0*.*70*	*0.63–0.78*	*<0*.*0001*
*STEMI*	*7*.*22*	*5.43–9.59*	*<0*.*0001*
*Smoking*	*0*.*66*	*0.60–0.73*	*<0*.*0001*
*Dyslipidemia*	*0*.*69*	*0.62–0.75*	*<0*.*0001*
*Prior Myocardial Infarction*	*0*.*71*	*0.58–0.86*	*<0*.*01*
*Marfan Syndrome*	*0*.*42*	*0.23–0.76*	*<0*.*01*
*Diabetes*	*0*.*85*	*0.76–0.96*	*<0*.*01*
*Depression*	*0*.*79*	*0.67–0.93*	*0*.*01*
*Hypothyroidism*	*0*.*83*	*0.72–0.95*	*0*.*01*
*Valvular Disease*	*0*.*85*	*0.76–0.96*	*0*.*01*
*Chronic Kidney Disease*	*1*.*13*	*1.02–1.26*	*0*.*02*
*Anemia*	*0*.*89*	*0.81–0.98*	*0*.*02*
*Coronary Artery Disease*	*1*.*12*	*1.01–1.24*	*0*.*03*
*Chronic Lung Disease*	*1*.*12*	*1.01–1.25*	*0*.*04*
Liver Disease	1.21	1.00–1.46	0.05
Atrial Fibrillation	1.08	0.98–1.20	0.12
Obesity	0.90	0.78–1.04	0.15
Ehlers-Danlos Syndrome	2.39	0.58–9.92	0.23
Heart Failure	1.28	1.21–1.36	0.78
Drug Use Disorder	1.02	0.79–1.32	0.86
Alcohol Use Disorder	1.00	0.78–1.27	0.97

Italicized variables indicate statistical significance.

STEMI, ST elevation myocardial infarction.

^a^ Age was analyzed as a continuous variable; for every year increase, there is a 4% increase in odds of in-patient mortality.

### Readmission risk

**Figure 2:**
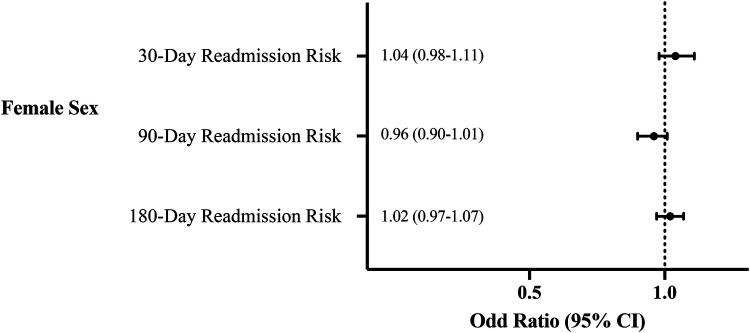
Multivariate analysis of the association of sex with readmission risk.

After adjusting for patient characteristics and comorbidities, female sex was not associated with increased risk of readmission at 30, 90, or 180 days (Figure 2).

## Discussion

This analysis, which to our knowledge constitutes the first nationally representative study to evaluate sex differences in in-hospital mortality and readmission risk in patients with TBAD who were managed medically, highlights several key findings. First, in patients with TBAD, women were older and had similar rates of hypertension than men, but an otherwise more favorable cardiovascular risk profile. Second, despite differences in atherosclerotic cardiovascular disease and atherosclerotic risk factors, as well as lower rates of complications during admission, women had higher rates of in-hospital mortality than men. Third, women had lower rates of elective admissions, shorter median LOS, and lower total charges than men. Fourth, while female sex was independently associated with increased odds of in-hospital mortality, there was no association between sex and risk of readmission at 30, 90, and 180 days, even after adjusting for in-hospital mortality.

Previous research has reasoned that sex differences in management and outcomes in patients with aortic dissections may be due to differences in cardiovascular risk profiles. For example, prior work has found that women tend to have more comorbidities, including higher rates of hypertension, diabetes, heart failure, chronic pulmonary disease, and rheumatologic conditions (3, 9). In this study, women with TBAD had a more favorable cardiovascular risk profile than men, yet had higher mortality. While hypertension and smoking are considered to be the main risk factors for thoracic aortic dissections (21, 22), we found that women had lower rates of smoking than men and there were no sex differences in rates of hypertension. Further research should focus on elucidating specific comorbidities that can help medical providers better risk stratify women and men with TBAD. For example, studies have investigated the role of thyroid hormones in acute aortic dissection outcomes (23, 24), and have shown that low T3 levels are associated with increased in-hospital mortality in patients with aortic dissection. Given the higher rates of hypothyroidism in women, including in our cohort, it would be important to better understand less obvious risk factors in acute aortic syndromes.

Our findings demonstrate that, while women with TBAD may not be at risk for readmission or increased in-hospital complications, they have worse immediate outcomes. Age may be one key factor in sex differences in in-hospital mortality. As was found in our study, association between older age and TBAD mortality has been well documented (25). Patients under the age of 70 years with complicated TBAD have decreased in-hospital mortality compared to those over the age of 70 years, regardless of management strategy (26). The older age at presentation in women may be attributed to the protective effects of female sex hormones during reproductive years (27). Aortic walls in women exhibit an upregulation of estrogen receptors, which confers a vasoprotective effect and reduces vessel inflammation (15, 28). However, this protection appears to diminish after menopause. Cheung et al. found that the expansion rates of degenerative thoracic aortic aneurysms were two to three times faster in women than in men, the majority of whom were postmenopausal (27). A rodent model of abdominal aorta aneurysm formation showed that estrogen inhibited matrix metalloproteinase (MMP)-9 production and aortic macrophage infiltration (29). Changes in aortic wall architecture, remodeling, and biomechanics in older, post-menopausal women may potentially be linked to the increased in-hospital mortality seen in women with TBAD.

It is similarly essential to highlight the differences in clinical presentations between men and women with TBAD. Patients with TBAD treated medically may be comparatively less symptomatic in relation to those with complicated aortic dissection who may experience the classic sequelae of malperfusion (25). In a review of patients with both Type A and TBAD, a lower proportion of women presented within six hours of symptom onset compared to men, and 40% of women waited over 24 h before first medical contact (3). This was explained by women experiencing less classic symptoms, decreased perception of pain, or having less frequent abrupt onset of symptoms and/or more frequently observed altered mental status (3)—features which may not only affect timing of hospital presentation, but may also result in diagnostic and treatment delay (2). In cases of TBAD, women are less likely to receive anti-hypertensive medications, including beta-blockers, ACE inhibitors, and angiotensin II receptor blockers compared to men (3, 30). It is also notable that women were more likely to be discharged to a skilled nursing facility or home with home health care in our study, suggesting that women are more likely to be sicker than men at time of discharge. Given that survival rates decrease with delays in presentation, diagnosis, and initiation of definitive treatment from symptom onset, public health and system-level strategies must be established to ensure timely intervention for patients with TBAD (31).

Anatomic and imaging-based differences must also be considered with respect to both sex differences and readmissions noted in this study. There are limited data regarding imaging findings for patients with TBAD managed medically. Nienaber et al. found no sex-differences in the number or type of diagnostic imaging studies used for patients with aortic dissections (3). It is known that women may suffer from complications associated with abdominal aortic aneurysms at smaller diameters than men (32). O'Donnell et al. showed that among patients with abdominal aortic aneurysms who undergo endovascular aortic repair, women had higher rates of long-term Type 1A endoleaks, which could in part be attributed to challenging anatomy, including more angulated necks (32). A study of patients with TBAD found that at time of diagnosis, women more commonly had evidence of a DeBakey type IIIa dissection, or distal aortic dissection ending above the level of the diaphragm, than men (5). Women also had a higher proportion of intramural hematoma, which could potentially explain a lower rate of end-organ malperfusion or lower-extremity ischemia than in men (5, 33), and coupled with delays in diagnosis, older age, and varied symptoms, could explain the worse acute outcomes in women, but absence of significant differences in readmissions amongst both sexes.

To our knowledge, this is the first nationally representative study to evaluate readmission risk in patients with TBAD who are managed medically, as prior studies have focused on patients with TBAD who undergo procedures. Treffalls and colleagues found that in patients who underwent repair of a TBAD, female sex was not associated with a one year risk of readmission, although this study did not stratify baseline demographics or comorbidities by sex (34). Another study showed that female sex was independently associated with reduced risk of 30-day readmission after TEVAR (10), and men were significantly more likely to have post-TEVAR arrhythmias, pneumonia, respiratory failure, AKI, stroke, and sepsis. A study of patients with TBAD managed both invasively and medically in Florida and New York found that female sex was associated with an increased risk of 30-day readmission but not with readmission risk at 90 days or two years for unspecified reasons (35).

This study should be interpreted in the context of certain limitations. First, we identified diagnoses and procedures using ICD-10-CM codes, which portends a risk of misclassification. Second, the use of ICD-10-CM codes also does not allow for obtaining important clinical characteristics such timing, utilization, and optimization of medical therapy, mortality at higher volume aortic centers vs. community-based settings, and imaging and anatomic features which limits our ability to assess sex differences in these factors. Third, the NRD does not include data on race and ethnicity which may play a contributory role given the important intersection between race and sex. Relatedly, multiple studies have shown disparities in cardiovascular care and outcomes among Black, Hispanic, and Native American patients (36–38). Fourth, this data set does not differentiate between sex, gender, and gender identity. Fifth, laboratory values are not included in the NRD, which may further delineate the severity of dissection. Lastly, out of hospital mortality is not assessed, which can theoretically impact readmission rates.

## Conclusion

To our knowledge, this is the first nationally representative study to evaluate sex differences in outcomes and readmission risk in patients with medically managed TBAD. Although women had lower rates of atherosclerotic disease and risk factors, they had higher in-hospital mortality compared to men. However, there was no difference in readmission risk between the two sexes. Sex-specific differences in patient characteristics, symptoms, and delays in diagnosis and treatment may contribute to the poorer immediate outcomes in women. Future studies should focus on identifying causes of higher short-term mortality in women, including condition- (imaging and anatomic features), medication-, and hospital-specific factors.

## Data Availability

The original contributions presented in the study are included in the article/Supplementary Material, and further inquiries can be directed to the corresponding author.
