# Supplementary material for: Sex differences in hospital outcomes of medically-managed type B aortic dissection
Source: Front Cardiovasc Med. 2025 May 8;12:1597266. doi: 10.3389/fcvm.2025.1597266 (PMC12095156; doi:10.3389/fcvm.2025.1597266)
Supplement: Supplementary file 1 [file Table1.docx]

**Supplementary Table S1—International Classification of Disease Codes Utilized in This Study**

| **Inclusion Criteria** | **ICD-10-CM Codes** |
| --- | --- |
| Dissection of thoracic aorta | I7101 |
| Dissection of thoracoabdominal aorta | I7103 |
| Dissection of unspecified site of aorta | I7100 |
| **Exclusion Criteria** | **ICD-10-CM Codes** |
| Cardioplegia | 3E080GC, 3E083GC |
| Valve Repair | 02NF3ZZ, 02NF4ZZ, 02NG3ZZ, 02NG4ZZ, 02NH3ZZ, 02NH4ZZ, 02NJ3ZZ, 02NJ4ZZ, 02RF37Z, 02RF38Z, 02RF3JZ, 02RF3KZ, 02RF37H, 02RF38H, 02RF3JH, 02RF3KH, 02RH37Z, 02RH38Z, 02RH3JZ, 02RH3KZ, 02RH37H, 02RH38H, 02RH3JH, 02RH3KH, 02RG37H, 02RG37Z, 02RG38H, 02RG38Z, 02RG3JH, 02RG3JZ, 02RG3KH, 02RG3KZ, 02RJ37H, 02RJ37Z, 02RJ38H, 02RJ38Z, 02RJ3JH, 02RJ3JZ, 02RJ3KH, 02RJ3KZ, 02QF0ZZ, 02QG0ZZ, 02QH0ZZ, 02QJ0ZZ, 027F04Z, 027F0DZ, 027F0ZZ, 02NF0ZZ, 027G04Z, 027G0DZ, 027G0ZZ, 02NG0ZZ, 02VG0ZZ, 027H04Z, 027H0DZ, 027H0ZZ, 027J04Z, 027J0DZ, 027J0ZZ, 02NJ0ZZ, 02RF07Z, 02RF08Z, 02RF0JZ, 02RF0KZ, 02RF47Z, 02RF48Z, 02RF4JZ, 02RF4KZ, 02RG07Z, 02RG08Z, 02RG0JZ, 02RG0KZ, 02RG47Z, 02RG48Z, 02RG4JZ, 02RG4KZ, 02RH07Z, 02RH08Z, 02RH0JZ, 02RH0KZ, 02RH47Z, 02RH48Z, 02RH4JZ, 02RH4KZ, 02RJ07Z, 02RJ08Z, 02RJ0JZ, 02RJ0KZ, 02RJ47Z, 02RJ48Z, 02RJ4JZ, 02RJ4KZ, 028D0ZZ, 028D3ZZ, 028D4ZZ, 02QD0ZZ, 02QD3ZZ, 02QD4ZZ, 02890ZZ, 02893ZZ, 02894ZZ, 02Q90ZZ, 02Q93ZZ, 02Q94ZZ, 02QF3ZZ, 02QF4ZZ, 02QG3ZZ, 02QG4ZZ, 02QH3ZZ, 02QH4ZZ, 02QJ3ZZ, 02QJ4ZZ, 024G0J2, 024J0J2, 024G072, 024G082, 024G0K2, 024J072, 024J082, 024J0K2, 024F07J, 024F08J, 024F0JJ, 024F0KJ, 02UG3JZ, 02NH0ZZ, 02UF07J, 02UF07Z, 02UF08J, 02UF08Z, 02UF0JJ, 02UF0JZ, 02UF0KJ, 02UF0KZ, 02UF47J, 02UF47Z, 02UF48J, 02UF48Z, 02UF4JJ, 02UF4JZ, 02UF4KJ, 02UF4KZ, X2RF032, X2RF432, 02UF37J, 02UF37Z, 02UF38J, 02UF38Z, 02UF3JJ, 02UF3JZ, 02UF3KJ, 02UF3KZ, X2RF332, 02UG07E, 02UG07Z, 02UG08E, 02UG08Z, 02UG0JE, 02UG0JZ, 02UG0KE, 02UG0KZ, 02UG47E, 02UG47Z, 02UG48E, 02UG48Z, 02UG4JE, 02UG4JZ, 02UG4KE, 02UG4KZ, 02UH07Z, 02UH08Z, 02UH0JZ, 02UH0KZ, 02UH47Z, 02UH48Z, 02UH4JZ, 02UH4KZ, 02UJ07G, 02UJ07Z, 02UJ08G, 02UJ08Z, 02UJ0JG, 02UJ0JZ, 02UJ0KG, 02UJ0KZ, 02UJ47G, 02UJ47Z, 02UJ48G, 02UJ48Z, 02UJ4JG, 02UJ4JZ, 02UJ4KG, 02UJ4KZ, 02UG37E, 02UG37Z, 02UG38E, 02UG38Z, 02UG3JE, 02UG3JH, 02UG3KE, 02UG3KZ, 02UH38Z, 02UH37Z, 02UH3JZ, 02UH3KZ, 02UJ37G, 02UJ37Z, 02UJ38G, 02UJ38Z, 02UJ3JG, 02UJ3JZ, 02UJ3KG, 02UJ3KZ, 02TH0ZZ, 02TH4ZZ, 02TH3ZZ, 027F3ZZ, 027F4ZZ, 027G3ZZ, 027G4ZZ, 027H3ZZ, 027H4ZZ, 027J3ZZ, 027J4ZZ, 027H4DZ, 027H44Z, 027G4DZ, 027G44Z, 027F4DZ, 027F44Z, 027J44Z, 027J4DZ, 027F34Z, 027F3DZ, 027G34Z, 027G3DZ, 027H34Z, 027H3DZ, 027J34Z, 027J3DZ, 02CF0ZZ, 02CF4ZZ, 02CG0ZZ, 02CG4ZZ, 02CH0ZZ, 02CH4ZZ, 02CJ0ZZ, 02CJ4ZZ, 02QF0ZJ, 02QF3ZJ, 02QF4ZJ, 02QG0ZE, 02QG3ZE, 02QG4ZE, 02QJ0ZG, 02QJ3ZG, 02QJ4ZG, 02VG3ZZ, 02VG4ZZ, 025J4ZZ, 025J0ZZ, 025H4ZZ, 025H0ZZ, 025G4ZZ, 025G0ZZ, 025F4ZZ, 025F0ZZ, 02RH38L, 02RH38M, 02BK0ZZ, 02BK3ZZ, 02BK4ZZ, 02NK0ZZ, 02NK3ZZ, 02NK4ZZ, 02NL0ZZ, 02NL3ZZ, 02NL4ZZ, 02QA0ZZ, 02QA3ZZ, 02QA4ZZ, 02B50ZZ, 02B53ZZ, 02B54ZZ, 02RM0JZ, 02RM4JZ, 02U50JZ, 02U53JZ, 02U54JZ, 02UM0JZ, 02UM3JZ, 02UM4JZ, 02RM07Z, 02RM0KZ, 02RM47Z, 02RM4KZ, 02U507Z, 02U508Z, 02U50KZ, 02U537Z, 02U538Z, 02U53KZ, 02U547Z, 02U548Z, 02U54KZ, 02UM07Z, 02UM0KZ, 02UM37Z, 02UM3KZ, 02UM47Z, 02UM4KZ, 02UM38Z, 02UM48Z, 02RK07Z, 02RK0KZ, 02RK47Z, 02RK4KZ, 02RL07Z, 02RL0KZ, 02RL47Z, 02RL4KZ, 02U607Z, 02U608Z, 02U707Z, 02U708Z, 02U70KZ, 02U737Z, 02U738Z, 02U73KZ, 02U747Z, 02U748Z, 02U74KZ, 02UK0KZ, 02UK3KZ, 02UK4KZ, 02UL0KZ, 02UL3KZ, 02UL4KZ, 02Q50ZZ, 02Q53ZZ, 02Q54ZZ, 02QM0ZZ, 02QM3ZZ, 02QM4ZZ, 02QB0ZZ, 02QB3ZZ, 02QB4ZZ, 02QC0ZZ, 02QC3ZZ, 02QC4ZZ, 021708S, 021708T, 021708U, 021709S, 021709T, 021709U, 02170AS, 02170AT, 02170AU, 02170JS, 02170JT, 02170JU, 02170KS, 02170KT, 02170KU, 02170ZS, 02170ZT, 02170ZU, 021748S, 021748T, 021748U, 021749S, 021749T, 021749U, 02174AS, 02174AT, 02174AU, 02174JS, 02174JT, 02174JU, 02174KS, 02174KT, 02174KU, 02174ZS, 02174ZT, 02174ZU, 021V08S, 021V08T, 021V08U, 021V09S, 021V09T, 021V09U, 021V0AS, 021V0AT, 021V0AU, 021V0JS, 021V0JT, 021V0JU, 021V0KS, 021V0KT, 021V0KU, 021V0ZS, 021V0ZT, 021V0ZU, 021V48S, 021V48T, 021V48U, 021V49S, 021V49T, 021V49U, 021V4AS, 021V4AT, 021V4AU, 021V4JS, 021V4JT, 021V4JU, 021V4KS, 021V4KT, 021V4KU, 021V4ZS, 021V4ZU, 02S00ZZ, 02S10ZZ, 02SP0ZZ, 021K08P, 021K08Q, 021K08R, 021K09P, 021K09Q, 021K09R, 021K0AP, 021K0AQ, 021K0AR, 021K0JP, 021K0JQ, 021K0JR, 021K0KP, 021K0KQ, 021K0KR, 021K0ZP, 021K0ZQ, 021K0ZR, 021K48P, 021K48Q, 021K48R, 021K49P, 021K49Q, 021K49R, 021K4AP, 021K4AQ, 021K4AR, 021K4JP, 021K4JQ, 021K4JR, 021K4KP, 021K4KQ, 021K4KR, 021K4ZP, 021K4ZQ, 021K4ZR |
| Operation of vessels of the heart | 02700ZZ, 02710ZZ, 02720ZZ, 02730ZZ, 02C00ZZ, 02C10ZZ, 02C20ZZ, 02C30ZZ, 3E07017, 3E070PZ, 3E07317, 3E073PZ, 02C03ZZ, 02C04ZZ, 02C13ZZ, 02C14ZZ, 02C23ZZ, 02C24ZZ, 02C33ZZ, 02C34ZZ, 0210093, 02100A3, 02100J3, 02100K3, 02100Z3, 0210493, 02104A3, 02104J3, 02104K3, 02104Z3, 021008W, 021009W, 02100AW, 02100JW, 02100KW, 021048W, 021049W, 02104AW, 02104JW, 02104KW, 021108W, 021109W, 02110AW, 02110JW, 02110KW, 021148W, 021149W, 02114AW, 02114JW, 02114KW, 021208W, 021209W, 02120AW, 02120JW, 02120KW, 021248W, 021249W, 02124AW, 02124JW, 02124KW, 021308W, 021309W, 02130AW, 02130JW, 02130KW, 021348W, 021349W, 02134AW, 02134JW, 02134KW, 0210088, 0210089, 021008C, 0210098, 0210099, 021009C, 02100A8, 02100A9, 02100AC, 02100J8, 02100J9, 02100JC, 02100K8, 02100K9, 02100KC, 02100Z8, 02100Z9, 02100ZC, 0210488, 0210489, 021048C, 0210498, 0210499, 021049C, 02104A8, 02104A9, 02104AC, 02104J8, 02104J9, 02104JC, 02104K8, 02104K9, 02104KC, 02104Z8, 02104Z9, 02104ZC, 0211088, 0211089, 021108C, 0211098, 0211099, 021109C, 02110A8, 02110A9, 02110AC, 02110J8, 02110J9, 02110JC, 02110K8, 02110K9, 02110KC, 02110Z8, 02110Z9, 02110ZC, 0211488, 0211489, 021148C, 0211498, 0211499, 021149C, 02114A8, 02114A9, 02114AC, 02114J8, 02114J9, 02114JC, 02114K8, 02114K9, 02114KC, 02114Z8, 02114Z9, 02114ZC, 021208C, 021209C, 02120AC, 02120JC, 02120KC, 02120ZC, 021248C, 021249C, 02124AC, 02124JC, 02124KC, 02124ZC, 021308C, 021309C, 02130AC, 02130JC, 02130KC, 02130ZC, 021348C, 021349C, 02134AC, 02134JC, 02134KC, 02134ZC, 021008F, 021009F, 02100AF, 02100JF, 02100KF, 02100ZF, 021048F, 021049F, 02104AF, 02104JF, 02104KF, 02104ZF, 0210083, 0210093, 02100A3, 02100J3, 02100K3, 02100Z3, 0210483, 0210493, 02104A3, 02104J3, 02104K3, 02104Z3, 021K0Z5, 021L0Z5, 021K4Z5, 021L4Z5, 3E070GC, 3E073GC, 3E074GC, 3E080GC, 3E083GC, 3E084GC, 0210344, 02103D4, 0210444, 02104D4, 0211344, 02113D4, 0211444, 02114D4, 0212344, 02123D4, 0212444, 02124D4, 0213344, 02133D4, 0213444, 02134D4, 02Q00ZZ, 02Q03ZZ, 02Q04ZZ, 02Q40ZZ, 02Q43ZZ, 02Q44ZZ, 02H40YZ, 02H43YZ, 02H44YZ, 02N00ZZ, 02N03ZZ, 02N04ZZ, 02N10ZZ, 02N13ZZ, 02N14ZZ, 02N20ZZ, 02N23ZZ, 02N24ZZ, 02N30ZZ, 02N33ZZ, 02N34ZZ, 02QA4ZZ, 02QB4ZZ, 02QC4ZZ, 02QA3ZZ, 02QB3ZZ, 02QC3ZZ, 02N60ZZ, 02N63ZZ, 02N64ZZ, 02N70ZZ, 02N73ZZ, 02N74ZZ, 02NK0ZZ, 02NK3ZZ, 02NK4ZZ, 02NL0ZZ, 02NL3ZZ, 02NL4ZZ, 4A02X4Z, 4A02XFZ, 02B40ZX, 02B43ZX, 02B44ZX, 02B50ZX, 02B53ZX, 02B54ZX, 02B60ZX, 02B63ZX, 02B64ZX, 02B70ZX, 02B73ZX, 02B74ZX, 02B80ZX, 02B83ZX, 02B84ZX, 02B90ZX, 02B93ZX, 02B94ZX, 02BD0ZX, 02BD3ZX, 02BD4ZX, 02BF0ZX, 02BF3ZX, 02BF4ZX, 02BG0ZX, 02BG3ZX, 02BG4ZX, 02BH0ZX, 02BH3ZX, 02BH4ZX, 02BJ0ZX, 02BJ3ZX, 02BJ4ZX, 02BK0ZX, 02BK3ZX, 02BK4ZX, 02BL0ZX, 02BL3ZX, 02BL4ZX, 02BM0ZX, 02BM3ZX, 02BM4ZX, 02BF0ZZ, 02BF4ZZ, 02BG0ZZ, 02BG4ZZ, 02BH0ZZ, 02BH4ZZ, 02BJ0ZZ, 02BJ4ZZ, 02B60ZZ, 02B63ZZ, 02B64ZZ, 02B70ZZ, 02B73ZZ, 02B74ZZ, 02BK0ZZ, 02BK3ZZ, 02BK4ZZ, 02BL0ZZ, 02BL3ZZ, 02BL4ZZ, 02560ZZ, 02570ZZ, 025K0ZZ, 025L0ZZ, 02T80ZZ, 02563ZZ, 02573ZZ, 025K3ZZ, 025L3ZZ, 02570ZK, 02573ZK, 02574ZK, 02B70ZK, 02B73ZK, 02B74ZK, 02L70ZK, 02L73ZK, 02L74ZK, 02564ZZ, 02574ZZ, 025K4ZZ, 025L4ZZ, 02UA0JZ, 02UA3JZ, 02UA4JZ, 02163Z7, 02H60YZ, 02H63YZ, 02H64YZ, 02H70YZ, 02H73YZ, 02H74YZ, 02HA0YZ, 02HA3YZ, 02HA4YZ, 02HK0YZ, 02HK3YZ, 02HK4YZ, 02HL0YZ, 02HL3YZ, 02HL4YZ, 02YA0Z0, 02YA0Z1, 02YA0Z2, 02RK0JZ, 02RL0JZ, 02WA0JZ, 02PA0JZ, 02HA0RS, 02HA3RS, 02HA4RS, 5A02116, 5A02216, 5A02110, 5A02210, 02HA0RZ, 02HA3RZ, 02HA4RZ, 02WA0QZ, 02WA0RS, 02WA0RZ, 02WA3QZ, 02WA3RS, 02WA3RZ, 02WA4QZ, 02WA4RS, 02WA4RZ, 02PA0QZ, 02PA0RS, 02PA0RZ, 02PA3QZ, 02PA3RZ, 02PA4QZ, 02PA4RS, 02PA4RZ, 02HA0QZ, 02HA3QZ, 02HA4QZ, 02HA0RJ, 02HA3RJ, 02HA4RJ, 5A0221D, 02L70CK, 02L70DK, 02L73CK, 02L73DK, 02L74CK, 02L74DK |
| Aortic Rupture | I711, I713, I715 |
| Dissection- carotid artery | I7771 |
| Dissection- vertebral artery | I7774 |
| Pericardial effusion | 02CN0ZZ, 02CN3ZZ, 02CN4ZZ, 02NN0ZZ, 02NN3ZZ, 02NN4ZZ, 0W9D00Z, 0W9D0ZX, 0W9D0ZZ, 0WCD0ZZ, 0WCD3ZZ, 0WCD4ZZ, 02BN0ZX, 02BN3ZX, 02BN4ZX, 02BN0ZZ, 02BN3ZZ, 02BN4ZZ, 02TN0ZZ, 02TN3ZZ, 02TN4ZZ, 0W9C30Z, 0W9C3ZZ, 0W9D30Z, 0W9D3ZX, 0W9D3ZZ, 0W9D40Z, 0W9D4ZX, 0W9D4ZZ, I314, 025N0ZZ, 025N3ZZ, 025N4ZZ, 02BN0ZZ, 02BN3ZZ, 02BN4ZZ, 02FN0ZZ, 02FN3ZZ, 02FN4ZZ, 02FNXZZ, 02NN0ZZ, 02NN3ZZ, 02NN4ZZ, 02TN0ZZ, 02TN3ZZ, 02TN4ZZ, 0W2DX0Z, 0W3D3ZZ, 0WPD30Z, 0WPDX0Z, 0WWD30Z, 0WWDX0Z, 3E1Y38X, 3E1Y38Z, 02JA3ZZ, 0WJD0ZZ, 0WJD3ZZ, 02HN0YZ, 02HN3YZ, 02HN4YZ, 02QN0ZZ, 02QN3ZZ, 02QN4ZZ, I319 |
| Thoracic Endovascular Aortic Repair | 02UW3JZ, 02UW4JZ, 02VW0DZ, 02VW3DZ, 02VW4DZ |
| Open Repair | 02BW0ZZ, 02BW4ZZ,02BX0ZZ, 02BX4ZZ, 04B00ZZ, 04B04ZZ, 02BP0ZZ, 02BP4ZZ, 02BQ0ZZ, 02BQ4ZZ, 02BR0ZZ, 02BR4ZZ, 02BS0ZZ, 02BS4ZZ, 02BT0ZZ, 02BT4ZZ, 02BV0ZZ, 02BV4ZZ, 03B00ZZ, 03B04ZZ, 03B10ZZ, 03B14ZZ, 03B20ZZ, 03B24ZZ, 03B30ZZ, 03B34ZZ, 03B40ZZ, 03B44ZZ, 05B00ZZ, 05B04ZZ, 05B10ZZ, 05B14ZZ, 05B30ZZ, 05B34ZZ, 05B40ZZ, 05B44ZZ, 05B50ZZ, 05B54ZZ, 05B60ZZ, 05B64ZZ, 04R007Z, 04R00JZ, 04R00KZ, 04R047Z, 04R04JZ, 04R04KZ, 02RP07Z, 02RP08Z, 02RP0JZ, 02RP0KZ, 02RP47Z, 02RP48Z, 02RP4JZ, 02RP4KZ, 02RQ07Z, 02RQ08Z, 02RQ0JZ, 02RQ0KZ, 02RQ47Z, 02RQ48Z, 02RQ4JZ, 02RQ4KZ, 02RR07Z, 02RR08Z, 02RR0JZ, 02RR0KZ, 02RR47Z, 02RR48Z, 02RR4JZ, 02RR4KZ, 02RS07Z, 02RS08Z, 02RS0JZ, 02RS0KZ, 02RS47Z, 02RS48Z, 02RS4JZ, 02RS4KZ, 02RT07Z, 02RT08Z, 02RT0JZ, 02RT0KZ, 02RT47Z, 02RT48Z, 02RT4JZ, 02RT4KZ, 02RV07Z, 02RV08Z, 02RV0JZ, 02RV0KZ, 02RV47Z, 02RV48Z, 02RV4JZ, 02RV4KZ, 02RW07Z, 02RW08Z, 02RW0JZ, 02RW0KZ, 02RW47Z, 02RW48Z, 02RW4JZ, 02RW4KZ, 02RX07Z, 02RX08Z, 02RX0JZ, 02RX0KZ, 02RX47Z, 02RX48Z, 02RX4JZ, 02RX4KZ, 03R007Z, 03R00JZ, 03R00KZ, 03R047Z, 03R04JZ, 03R04KZ, 03R107Z, 03R10JZ, 03R10KZ, 03R147Z, 03R14JZ, 03R14KZ, 03R207Z, 03R20JZ, 03R20KZ, 03R247Z, 03R24JZ, 03R24KZ, 03R307Z, 03R30JZ,03R30KZ, 03R347Z, 03R34JZ, 03R34KZ, 03R407Z, 03R40JZ, 03R40KZ, 03R447Z, 03R44JZ, 03R44KZ, 05R007Z, 05R00JZ, 05R00KZ, 05R047Z, 05R04JZ, 05R04KZ, 05R107Z, 05R10JZ, 05R10KZ, 05R147Z, 05R14JZ, 05R14KZ, 05R307Z, 05R30JZ, 05R30KZ, 05R347Z, 05R34JZ, 05R34KZ, 05R407Z, 05R40JZ, 05R40KZ, 05R447Z, 05R44JZ, 05R44KZ, 05R507Z, 05R50JZ, 05R50KZ, 05R547Z, 05R54JZ, 05R54KZ, 05R607Z, 05R60JZ, 05R60KZ, 05R647Z, 05R64JZ, 05R64KZ, 02UP0JZ, 02UP3JZ, 02UP4JZ, 02UQ0JZ, 02UQ3JZ, 02UQ4JZ, 02UR0JZ, 02UR3JZ, 02UR4JZ, 02US0JZ, 02US3JZ, 02US4JZ, 02UT0JZ, 02UT3JZ, 02UT4JZ, 02UX0JZ, 03U00JZ, 03U03JZ, 03U04JZ, 03U10JZ, 03U13JZ, 03U14JZ, 03U20JZ, 03U23JZ, 03U24JZ, 03U30JZ, 03U33JZ, 03U34JZ, 03U40JZ, 03U43JZ, 03U44JZ, 03U50JZ, 03U53JZ, 03U54JZ, 03U60JZ, 03U63JZ, 03U64JZ, 03U70JZ, 03U73JZ, 03U74JZ, 03U80JZ, 03U83JZ, 03U84JZ, 03U90JZ, 03U93JZ, 03U94JZ, 03UA0JZ, 02UW0JZ, 02UV4JZ, 02UV3JZ, 02UV0JZ, 03UA3JZ, 03UA4JZ, 03UB0JZ, 03UB3JZ, 03UB4JZ, 03UC0JZ, 03UC3JZ, 03UC4JZ, 03UD0JZ, 03UD3JZ, 03UD4JZ, 03UF0JZ, 03UF3JZ, 03UF4JZ, 03UG0JZ, 03UG3JZ, 03UG4JZ, 03UH0JZ, 03UH3JZ, 03UH4JZ, 03UJ0JZ, 03UJ3JZ, 03UJ4JZ, 03UK0JZ, 03UK3JZ, 03UK4JZ, 03UL0JZ, 03UL3JZ, 03UL4JZ, 03UM0JZ, 03UM3JZ, 03UM4JZ, 03UN0JZ, 03UN3JZ, 03UN4JZ, 03UP0JZ, 03UP3JZ, 03UP4JZ, 03UQ0JZ, 03UQ3JZ, 03UQ4JZ, 03UR0JZ, 03UR3JZ, 03UR4JZ, 03US0JZ, 03US3JZ, 03US4JZ, 03UT0JZ, 03UT3JZ, 03UT4JZ, 03UU0JZ, 03UU3JZ, 03UU4JZ, 03UV0JZ, 03UV3JZ, 03UV4JZ, 03UY0JZ, 03UY3JZ, 03UY4JZ, 04U00JZ, 04U03JZ, 04U04JZ, 04U10JZ, 04U13JZ, 04U14JZ, 04U20JZ, 04U23JZ, 04U24JZ, 04U30JZ, 04U33JZ, 04U34JZ, 04U40JZ, 04U43JZ, 04U44JZ, 04U50JZ, 04U53JZ, 04U54JZ, 04U60JZ, 04U63JZ, 04U64JZ, 04U70JZ, 04U73JZ, 04U74JZ, 04U80JZ, 04U83JZ, 04U84JZ, 04U90JZ, 04U93JZ, 04U94JZ, 04UA0JZ, 04UA3JZ, 04UA4JZ, 04UB0JZ, 04UB3JZ, 04UB4JZ, 04UC0JZ, 04UC3JZ, 04UC4JZ, 04UD0JZ, 04UD3JZ, 04UD4JZ, 04UE0JZ, 04UE3JZ, 04UE4JZ, 04UF0JZ, 04UF3JZ, 04UF4JZ, 04UH0JZ, 04UH3JZ, 04UH4JZ, 04UJ0JZ, 04UJ3JZ, 04UJ4JZ, 04UK0JZ, 04UK3JZ, 04UK4JZ, 04UL0JZ, 04UL3JZ, 04UL4JZ, 04UM0JZ, 04UM3JZ, 04UM4JZ, 04UN0JZ, 04UN3JZ, 04UN4JZ, 04UP0JZ, 04UP3JZ, 04UP4JZ, 04UQ0JZ, 04UQ3JZ, 04UQ4JZ, 04UR0JZ, 04UR3JZ, 04UR4JZ, 04US0JZ, 04US3JZ, 04US4JZ, 04UT0JZ, 04UT3JZ, 04UT4JZ, 04UU0JZ, 04UU3JZ, 04UU4JZ, 04UV0JZ, 04UV3JZ, 04UV4JZ, 04UW0JZ, 04UW3JZ, 04UW4JZ, 04UY0JZ, 04UY3JZ, 04UY4JZ, 05U00JZ, 05U03JZ, 05U04JZ, 05U10JZ, 05U13JZ, 05U14JZ, 05U30JZ, 05U33JZ, 05U34JZ, 05U40JZ, 05U43JZ, 05U44JZ, 05U50JZ, 05U53JZ, 05U54JZ, 05U60JZ, 05U63JZ, 05U64JZ, 05U70JZ, 05U73JZ, 05U74JZ, 05U80JZ, 05U83JZ, 05U84JZ, 05U90JZ, 05U93JZ, 05U94JZ, 05UA0JZ, 05UA3JZ, 05UA4JZ, 05UB0JZ, 05UB3JZ, 05UB4JZ, 05UC0JZ, 05UC3JZ, 05UC4JZ, 05UD0JZ, 05UD3JZ, 05UD4JZ, 05UF0JZ, 05UF3JZ, 05UF4JZ, 05UG0JZ, 05UG3JZ, 05UG4JZ, 05UH0JZ, 05UH3JZ, 05UH4JZ, 05UL0JZ, 05UL3JZ, 05UL4JZ, 05UM0JZ, 05UM3JZ, 05UM4JZ, 05UN0JZ, 05UN3JZ, 05UN4JZ, 05UP0JZ, 05UP3JZ, 05UP4JZ, 05UQ0JZ, 05UQ3JZ, 05UQ4JZ, 05UR0JZ, 05UR3JZ, 05UR4JZ, 05US0JZ, 05US3JZ, 05US4JZ, 05UT0JZ, 05UT3JZ, 05UT4JZ, 05UV0JZ, 05UV3JZ, 05UV4JZ, 05UY0JZ, 05UY3JZ, 05UY4JZ, 06U00JZ, 06U03JZ, 06U04JZ |
| Thoracic aortic aneurysm, without rupture | I712 |
| **Variables** | **ICD-10-CM Codes** |
| Hyperlipidemia | E780, E7800, E7801, E781, E782, E783, E784, E7841, E7849, E785 |
| Smoking | F17200, F17201, F17203, F17208, F17209, F17210, F17211, F17213, F17218, F17219, F17220, F17221, F17223, F17228, F17229, F17290, F17291, F17293, F17298, F17299, O99330, O99331, O99332, O99333, O99334, O99335, Z720, Z87891, Z5301, T65211A, T65211D, T65211S, T65212A, T65212D, T65212S, T65213A, T65213D, T65213S, T65214A, T65214D, T65214S, T65221A, T65221D, T65221S, T65222A, T65222D, T65222S, T65223A, T65223D, T65223S, T65224A, T65224D, T65224S, T65291A, T65291D, T65291S, T65292A, T65292D, T65292S, T65293A, T65293D, T65293S, T65294A, T65294D, T65294S |
| Anemia | D500, D501, D508, D509, D510, D511, D512, D513, D518, D519, D520, D521, D528, D529, D530, D531, D532, D538, D539, D550, D551, D552, D5521, D5529, D553, D558, D559, D560, D561, D562, D563, D564, D565, D568, D569, D580, D581, D582, D588, D589, D590, D591, D5910, D5911, D5912, D5913, D5919, D592, D593, D594, D595, D596, D598, D599, D600, D601, D608, D609, D6101, D6109, D611, D612, D613, D61810, D61811, D61818, D6182, D6189, D619, D62, D630, D631, D638, D640, D641, D642, D643, D644, D6481, D6489, D649 |
| Heart Failure | I110, I130, I132, I0981, I501, I5020, I5021, I5022, I5023, I5030, I5031, I5032, I5033, I5040, I5041, I5042, I5043, I50810, I50811, I50812, I50813, I50814, I5082, I5083, I5084, I5089, I509, I5181, A3681, B3324, D8685, I255, I420, I421, I422, I423, I424, I425, I426, I427, I428, I429, I43, O903 |
| Prior Myocardial Infarction | I252 |
| Valvular Disease | A1884, A3282, A3951, A5203, B3321, B376, I011, I018, I019, I020, I050, I051, I052, I058, I059, I060, I061, I062, I068, I069, I070, I071, I072, I078, I079, I080, I081, I082, I083, I088, I089, I091, I0989, I330, I339, I340, I341, I342, I348, I349, I350, I351, I352, I358, I359, I360, I361, I362, I368, I369, I370, I371, I372, I378, I379, I38, I39, M3211, Q220, Q221, Q222, Q223, Q224, Q225, Q226, Q228, Q229, Q230, Q231, Q232, Q233, Q234, Q238, Q239, T8201XA, T8201XD, T8201XS, T8202XA, T8202XD, T8202XS, T8203XA, T8203XD, T8203XS, T8209XA, T8209XD, T8209XS, T82221A, T82221D, T82221S, T82222A, T82222D, T82222S, T82223A, T82223D, T82223S, T82228A, T82228D, T82228S, T826XXA, T826XXD, T826XXS, Z952, Z953, Z954 |
| Coronary Artery Disease | I200, I201, I208, I209, I2510, I25110, I25111, I25118, I25119, I252, I255, I256, I25700, I25701, I25708, I25709, I25710, I25711, I25718, I25719, I25720, I25721, I25728, I25729, I25730, I25731, I25738, I25739, I25750, I25751, I25758, I25759, I25760, I25761, I25768, I25769, I25790, I25791, I25798, I25799, I25810, I25811, I25812, I2582, I2583, I2584, I2589, I259, Z951, Z955, Z9861, T82211D, T82211S, T82212D, T82212S, T82213D, T82213S, T82218D, T82218S, T82855D, T82855S, I252, I2101, I2102, I2109, I2111, I2119, I2121, I2129, I213, I220, I221, I228, I229, I214, I219, I21A1, I21A9, I222, Z951, I25700, I25701, I25708, I25709, I25710, I25711, I25718, I25719, I25720, I25721, I25728, I25729, I25730, I25731, I25738, I25739, I25760, I25761, I25768, I25769, I25790, I25791, I25798, I25799, I25810, I25812, T82211D, T82211S, T82212D, T82212S, T82213D, T82213S, T82218D, T82218S, Z955, Z9861, T82855A, I230, I231, I232, I233, I234, I235, I236, I237, I238, I240, I248, I249, T82211A, T82212A, T82213A, T82218A, T82855A |
| ST-segment Elevation Myocardial Infarction | I2101, I2102, I2109, I2111, I2119, I2121, I2129, I213, I220, I221, I228, I229 |
| Atrial Fibrillation/ Flutter | I480, I481, I4811, I4819, I482, I4820, I4821, I483, I484, I4891, I4892 |
| Liver Disease | B190, B1911, B1921, I8500, I8501, I8510, I8511, I864, K7040, K7041, K7210, K7211, K7290, K7291, K765, K766, K767, K9182, A5145, A5274, B180, B181, B182, B188, B189, B1910, B1920, B199, B251, B581, K700, K7010, K7011, K702, K7030, K7031, K709, K713, K714, K7150, K7151, K716, K717, K718, K730, K731, K732, K738, K739, K740, K7400, K7401, K7402, K741, K742, K743, K744, K745, K7460, K7469, K751, K752, K753, K754, K7581, K7589, K759, K760, K761, K762, K763, K764, K7681, K7689, K769, K77, T8642 |
| Chronic Kidney Disease/End-Stage Renal Disease | N181, N182, N183, N1830, N1831, N1832, N184, N185, N186, N189, N19, D631, E0822, E0922, E1022, E1122, E1322, I120, I129, I130, I1310, I1311, I132, O10211, O10212, O10213, O10219, O1022, O1023, O10311, O10312, O10313, O10319, O1032, O103, T8612, I953, R880, Z4901, Z4902, Z4931, Z4932, 5A1D00Z, 5A1D60Z, 5A1D70Z, 5A1D80Z, 5A1D90Z, 3E1M39Z, Z9115, Z992, Y841 |
| Marfan Syndrome | Q8740 |
| Ehlers-Danlos Syndrome | Q796 |
| Acute Kidney Injury | N170, N171, N172, N178, N179, N990 |
| Dialysis | I953, R880, Z4901, Z4902, Z4931, Z4932, 5A1D00Z, 5A1D60Z, 5A1D70Z, 5A1D80Z, 5A1D90Z, 3E1M39Z, T8241XA, T8242XA, T8243XA, T8249XA, T85611A, T85621A, T85631A, T85691A, T8571XA, Z992, Z9115, Z992, Y841 |
| Intubation/Mechanical Ventilation | 5A1935Z, 5A1945Z, 5A1955Z, J95850, J95851, J95859, Z9911, Z9912, Z930, J9500, J9501, J9502, J9503, J9504, J9509, Z430 |
| Cardiac Arrest | I462, I468, I469, I97120, I97121, I97710, I97711, 5A12012, 5A1221J, I4901 |
| Stroke/Transient Ischemic Accident | G9511, G450, G451, G452, G453, G454, G458, G459, G460, G461, G462, G463, G464, G465, G466, G467, G468, H3400, H3401, H3402, H3403, H3410, H3411, H3412, H3413, H34211, H34212, H34213, H34219, H34231, H34232, H34233, H34239, H93011, H93012, H93013, H93019, I6000, I6001, I6002, I6010, I6011, I6012, I602, I6020, I6021, I6022, I6030, I6031, I6032, I604, I6050, I6051, I6052, I606, I607, I608, I609, I610, I611, I612, I613, I614, I615, I616, I618, I619, I6200, I6201, I6202, I621, I629, I6300, I63011, I63012, I63013, I63019, I6302, I63031, I63032, I63033, I63039, I6309 , I6310, I63111, I63112, I63113, I63119, I6312, I63131, I63132, I63133, I63139, I6319, I6320, I63211, I63212, I63213, I63219, I6322, I63231, I63232, I63233, I63239, I6329, I6330, I63311, I63312, I63313, I63319, I63321, I63322, I63323, I63329, I63331, I63332, I63333, I63339, I63341, I63342, I63343, I63349, I6339, I6340, I63411, I63412, I63413, I63419, I63421, I63422, I63423, I63429, I63431, I63432, I63433, I63439, I63441, I63442, I63443, I63449, I6349, I6350, I63511, I63512, I63513, I63519, I63521, I63522, I63523, I63529, I63531, I63532, I63533, I63539, I63541, I63542, I63543, I63549, I6359, I636, I638, I6381, I6389, I639, I6781, I6782, I97810, I97811, I97820, I97821, G43601, G43609, G43611, G43619, R29701, R29702, R29703, R29704, R29705, R29706, R29707, R29708, R29709, R29710, R29711, R29712, R29713, R29714, R29715, R29716, R29717, R29718, R29719, R29720, R29721, R29722, R29723, R29724, R29725, R29726, R29727, R29728, R29729, R29730, R29731, R29732, R29733, R29734, R29735, R29736, R29737, R29738, R29739, R29740, R29741, R29742 |
